# PD65 How Methods Innovation In Health Technology Assessment Missed The Opportunity To Include Health Equity In The Value Puzzle

**DOI:** 10.1017/S0266462324003222

**Published:** 2025-01-07

**Authors:** Grammati Sarri, Amruta Radhakrishnan, Jeffrey Muir, Ipek Ozer Stillman

## Abstract

**Introduction:**

Reconstructing the value puzzle in health technology assessment (HTA) of new technologies is an ongoing discussion among different stakeholders. Little progress has been made toward consistently and transparently incorporating additional value elements, such as health equity, or moving the focus beyond the traditional value elements of clinical benefit and economic cost associated with the introduction of new technologies.

**Methods:**

The objective was to conduct a series of pragmatic reviews of recent HTA guidance documents, international organizations, and previous systematic reviews to answer the following research questions.

- Would increased familiarity with real-world evidence, advanced analytics, and expanded forms of economic modeling create the forum for HTA bodies to reconsider their processes and include health equity when assessing new products?
- Which methods innovations would facilitate changes in HTA methods and processes to consistently and transparently assess health equity value elements?

Results were documented and qualitatively synthesized by outlining missed opportunities and highlighting potential barriers to integrating equity-informed HTA processes.

**Results:**

Our findings were grouped into three main parts: HTA guidance summaries, trends summarized by key organizations (HTAi, ISPOR, and others), and peer reviewed publications. HTA bodies have increasingly emphasized health equity concerns and the importance of standardizing methods to support health equity considerations but have not recommended explicit quantitative methods. Our database search found previous systematic literature reviews explicitly referring to methods of integrating real-world evidence into comparative effectiveness assessments, whereas modeling techniques such as distributional, augmented, or cost-effectiveness analyses, and multicriteria decision-making can integrate health equity effects for both patients and healthcare systems.

**Conclusions:**

Our research showcases the gap between recognizing health equity as a missing element in HTA and incorporating methods to implement such considerations into real-life decision-making. Greater familiarity with health equity methods may move the discussion from “whether” to “how” additional value elements such as health equity can inform decision-making.

